# Species-Specific Expression of Growth-Regulatory Genes in 2 Anoles with Divergent Patterns of Sexual Size Dimorphism

**DOI:** 10.1093/iob/obac025

**Published:** 2022-08-09

**Authors:** Christian L Cox, Michael L Logan, Daniel J Nicholson, Albert K Chung, Adam A Rosso, W Owen McMillan, Robert M Cox

**Affiliations:** Florida International University, 11200 SW 8th St, Miami, FL 33199, USA; Smithsonian Tropical Research Institute, Amador Causeway, Panama City, Panama; Georgia Southern University, 1332 Southern Dr, Statesboro, GA 30458, USA; Smithsonian Tropical Research Institute, Amador Causeway, Panama City, Panama; University of Nevada Reno, 1664 N Virginia St, Reno, NV 89557, USA; Smithsonian Tropical Research Institute, Amador Causeway, Panama City, Panama; Queen Mary University, Mile End Rd, Bethnal Green, London E1 4NS, UK; University of Texas-Arlington, 701 S Nedderman Dr. Arlington, TX 76019, USA; Smithsonian Tropical Research Institute, Amador Causeway, Panama City, Panama; Georgia Southern University, 1332 Southern Dr, Statesboro, GA 30458, USA; University of Texas-Arlington, 701 S Nedderman Dr. Arlington, TX 76019, USA; Princeton University, Princeton, NJ 08544, USA; Georgia Southern University, 1332 Southern Dr, Statesboro, GA 30458, USA; Smithsonian Tropical Research Institute, Amador Causeway, Panama City, Panama; University of Virginia, Charlottesville, VA 22904, USA

## Abstract

Sexual size dimorphism is widespread in nature and often develops through sexual divergence in growth trajectories. In vertebrates, the growth hormone/insulin-like growth factor (GH/IGF) network is an important regulator of growth, and components of this network are often regulated in sex-specific fashion during the development of sexual size dimorphism. However, expression of the GH/IGF network is not well characterized outside of mammalian model systems, and the extent to which species differences in sexual size dimorphism are related to differences in GH/IGF network expression is unclear. To begin bridging this gap, we compared GH/IGF network expression in liver and muscle from 2 lizard congeners, one with extreme male-biased sexual size dimorphism (brown anole, *Anolis sagrei*)*,* and one that is sexually monomorphic in size (slender anole, *A. apletophallus*). Specifically, we tested whether GH/IGF network expression in adult slender anoles resembles the highly sex-biased expression observed in adult brown anoles or the relatively unbiased expression observed in juvenile brown anoles. We found that adults of the 2 species differed significantly in the strength of sex-biased expression for several key upstream genes in the GH/IGF network, including insulin-like growth factors 1 and 2. However, species differences in sex-biased expression were minor when comparing adult slender anoles to juvenile brown anoles. Moreover, the multivariate expression of the entire GH/IGF network (as represented by the first two principal components describing network expression) was sex-biased for the liver and muscle of adult brown anoles, but not for either tissue in juvenile brown anoles or adult slender anoles. Our work suggests that species differences in sex-biased expression of genes in the GH/IGF network (particularly in the liver) may contribute to the evolution of species differences in sexual size dimorphism.

## Introduction

Sexual dimorphism is widespread in nature and understanding the genomic and transcriptomic mechanisms that facilitate the evolution of sex-specific phenotypes is a major goal of integrative biology (Lande 1980; Fairbairn 1997; Wiens 1999; Fairbairn and Roff 2006; Chenoweth et al. 2008; Mank 2009; Reedy et al. 2019). Because males and females share an autosomal genome, the development of sexual dimorphism usually requires the sex-specific expression of shared autosomal genes (Ellegren and Parsch 2007; Mank 2009; Innocenti and Morrow 2010; Mank et al. 2010; Ingleby et al. 2015). Consequently, evolutionary shifts in the direction or magnitude of sexual dimorphism should be accompanied by changes in the expression of gene networks that underly sexually dimorphic traits. Body size is a polygenic trait that often differs between the sexes as the result of sex-specific growth trajectories (Andrews 1976; Stamps 1993; Stamps and Krishnan 1997; Badyaev 2002; Cox and John-Alder 2007; Cox et al. 2017b). These growth trajectories are governed at least in part by the growth hormone/insulin-like growth factor (GH/IGF) regulatory network (McGaugh et al. 2015). Hence, comparative studies of sex-specific expression of the GH/IGF network should provide insight into the mechanisms that underlie the development of sexual size dimorphism.

The GH/IGF network is a conserved endocrine signaling network in vertebrates that regulates growth, among other functions (Butler et al. 2002; Gahete et al. 2009). Growth hormone releasing hormone (GHRH) is secreted by the hypothalamus and binds to growth hormone releasing hormone receptors (GHRHR) in the pituitary gland to stimulate the release of growth hormone (GH) (Hall et al. 1986). GH circulates to target tissues (e.g., liver, muscle) and subsequently binds to growth hormone receptors (GHR) to stimulate production and secretion of insulin-like growth factors IGF-1 and IGF-2 (Duan et al. 2010). While the liver is the primary source of circulating IGF peptides, many other tissues of the body also express *IGF* transcripts (Butler et al. 2002). Insulin-like growth factors bind to IGF receptors (IGF1R, IGF2R) in target tissues to initiate cellular responses that often involve insulin signaling and the mTOR (mechanistic target of rapamycin) networks (Butler et al. 2002; Gahete et al. 2009; McGaugh et al. 2015). IGF binding proteins (IGFBPs) are also produced and secreted by the liver to modulate the bioactivity and longevity of IGF peptides in circulation (Clemmons 1993; Duan and Xu 2005; Duan et al. 2010; Allard and Duan 2018). The effects of IGF-2 are also mediated by IGF-2 binding proteins (IGF2BPs), which bind to mRNA and have diverse and less well understood cellular functions (Bell et al. 2013). Although the components of this regulatory network seem to be broadly conserved across vertebrates, relatively little is known about the tissue- and sex-specific expression of genes in the GH/IGF network outside of a few well-studied systems (McGaugh et al. 2015; Schwartz and Bronikowski 2016).

Comparative research on the expression and molecular evolution of the GH/IGF network during ontogeny suggests intriguing differences between classical mammalian model species and other vertebrates, including other mammals. In rodent models (mice and rats), *IGF-1* is primarily expressed during post-natal development to regulate growth and nutrition, while *IGF-2* is primarily expressed prenatally (Wolf et al. 1998; Constância et al. 2002; Yakar and Adamo 2012). However, research in humans (Supplementary Information in Fagerberg et al. 2014) and other vertebrates, including lizards and snakes, has revealed post-natal expression of both *IGF-1* and *IGF-2* in multiple tissues (McGaugh et al. 2015; Beatty and Schwartz 2020). Likewise, there are many IGFBPs in mammals, with a core set of six (IGFBP1-6) that serve an important role in IGF function (Clemmons 1993; Jones and Clemmons 1995; Hwa et al. 1999; Duan and Xu 2005; Duan et al. 2010; Allard and Duan 2018). However, the function of IGFBPs varies greatly among non-mammalian vertebrates (Haramoto et al. 2014; Garcia de la Serrana and Macqueen 2018), and predictions based upon molecular structure of IGFBP6 suggest that it can no longer efficiently bind IGFs in squamates (McGaugh et al. 2015). This previous work highlights the potential for interspecific variation in expression and function of GH/IGF genes, including across ontogeny. Additional comparative research on the expression of GH/IGF genes is thus needed for a more comprehensive understanding of the role of this network in the ontogeny and evolution of body size, including sexual size dimorphism.

Previous research suggests that sex-biased expression of the GH/IGF network can regulate the development of sexual size dimorphism. In rodent models (rat and mouse) with male-biased sexual size dimorphism, sex-biased patterns of GH secretion regulate divergent growth between the sexes (Lichanska and Waters 2008). For male-larger brown anole lizards (*Anolis sagrei*), expression of genes for *GHR*, insulin-like growth factors (*IGF-1* and *IGF-2*), and IGFR and binding proteins (*IGFBP1* and *IGFBP4*) is male-biased (i.e., transcript abundance is higher in males) and stimulated by testosterone (Cox et al. 2017b). Similarly, 17-α-methyl-testosterone mediates male-biased expression of *GH*, *IGF-1*, and *IGF-2* in the yellow catfish (*Pelteobagrus fulvidraco*), which exhibits male-biased sexual size dimorphism (Ma et al. 2016). However, testosterone reduces expression of hepatic *IGF-1* in a lizard species (*Sceloporus undulatus*) with female-biased sexual size dimorphism (Duncan et al. 2020). In brown anoles, expression of genes in the GH/IGF network varies based upon tissue type and developmental stage and diverges between the sexes as development progresses (Cox et al. 2017b; Beatty and Schwartz 2020). Altogether, this body of work implies that sex-biased expression of the GH/IGF network is often involved in the development of sexual dimorphism. However, previous work has not explicitly compared the expression of the GH/IGF network between closely related species that differ in their patterns of sexual dimorphism.

In this study, we compared sex differences in expression of the GH/IGF network between two species of *Anolis* lizard: the sexually dimorphic brown anole (*A.sagrei*), and the sexually monomorphic Panamanian slender anole (*Anolis apletophallus*). Brown anoles are sexually dimorphic in many traits (Sanger et al. 2013; Sanger et al. 2014; Reedy et al. 2016, Cox et al. 2017a), including body size, which exhibits extreme male-biased sexual dimorphism (Stamps 1999; Cox et al. 2015) that has been linked to sex-biased expression of genes in the GH/IGF network in the liver (Cox et al. 2017b). Here, we extend this work by characterizing expression of the GH/IGF network in brown anoles in both liver and muscle, and at different ages that span sexual monomorphism (juveniles) through the emergence of sexual dimorphism (adults). In contrast, slender anoles are sexually monomorphic in body size, although they are dimorphic in other traits such as dewlap size (Andrews 1976; Rosso et al. 2020; Logan et al. 2021). Expression of the GH/IGF network has not been characterized in slender anoles. We measured the expression of genes in the GH/IGF network in adult slender anoles to compare to both juvenile and adult brown anoles. We sequenced the transcriptomes of both liver and muscle tissues of brown and slender anoles to (1) characterize ontogenetic changes in the expression of the GH/IGF network in brown anoles, (2) compare patterns of sex bias in genes of the GH/IGF network between brown and slender anoles, and (3) characterize patterns of sex bias across two tissues, each of which are related to sexual dimorphism in body size. We predicted that sex bias in GH/IGF expression would increase with age in brown anoles, coincident with increases in sexual size dimorphism, and that expression of GH/IGF genes in the sexually monomorphic slender anoles would be more similar to patterns in juvenile brown anoles (prior to emergence of pronounced sex bias) than to adult brown anoles (after the emergence of pronounced sex bias).

## Materials and methods

### Study subjects

We sampled tissues from brown anoles from a breeding colony comprised the third-generation descendants of lizards collected from Great Exuma in The Bahamas (23.5066 °N, −75.7660 °W). Colony founders were collected under approval from the Bahamas Engineering, Science and Technology (BEST) Commission and the Bahamas Ministry of Agriculture and imported with permission of the US Fish and Wildlife Service. An advantage of using animals from a breeding colony is that we know the precise age of individuals. Lizards were housed at the University of Virginia in individual plastic cages (29 × 19 × 18 cm, Lee's Kritter Keeper, Lee's Aquarium and Pet Products, San Marcos, CA, USA). Each cage contained a PVC pipe, a fiberglass screen hammock, and a potted plant. Cages were housed on shelving that was equipped with fluorescent light fixtures with two Reptisun 10.0 UVB Bulbs (Zoomed, San Luis Obispo, CA, USA) and maintained on a 12L:12D light schedule. Animals were maintained in the colony at 29°C and 65% relative humidity and fed crickets three times per week (dusted with Fluker's Calcium with Vitamin D3 and Fluker's Reptile Vitamin with Beta Carotene; Fluker's, Port Allen, LA, USA). Lizards were provided with drinking water by misting the cages daily.

Slender anoles were captured from the wild from central Panama in Soberania National Park (9.1344 °N, −79.7221 °W) by hand or using a lizard catchpole. The lizard catchpole consisted of a fiberglass fishing rod that had a loop tied with a braided fishing line at the top eyelet. Following capture, lizards were transported to the Gamboa Laboratory at the Smithsonian Tropical Research Institute in Gamboa, Panama. Lizards were then housed in plastic containers with a moistened paper towel and maintained in a temperature-controlled room (28°C) for 48 h on a 12L:12D light cycle prior to tissue collection. Slender anoles were collected under field collection permits issues by MiAmbiente of Panama.

### Tissue collection

We selected brown anoles from the colony at three different age points (4, 8, and 12 months post-hatching, *n* = 5 of each sex at each age point). We selected these time points because they represent (1) a point at which sexual dimorphism in size is just starting to develop (juveniles, 4 months), (2) a point at which growth is divergent between the sexes (subadults, 8 months), and (3) a point when animals are reproductively mature and sexual size dimorphism is near maximal levels (adults, 12 months of age). We sampled reproductively mature slender anoles (>38 mm SVL, *n* = 8 adults of each sex) directly from the wild for tissue sampling, which should be comparable to the adult (12 months) brown anoles in growth trajectories (Andrews and Rand 1974; Andrews 1979; Andrews et al. 1989). One caveat of not sampling juveniles in *A. apletophallus* is that we do not have a perfectly balanced design to test whether changes in sex-biased expression of the GH/IGF network are less pronounced across ontogeny in this species. However, the key prediction is that this would result in an adult transcriptome in *A. apletophallus* that is more similar to *A. sagrei* juveniles than to *A. sagrei* adults*,* and this key prediction can still be directly tested with our sampling design. For both species, lizards were fasted for a minimum of 48 h prior to sampling tissues. For brown anoles, lizards were removed from their cages immediately prior to tissue collection. For the slender anole, lizards were moved to an incubator maintained at 28°C for two h prior to sampling tissues. We focused on expression of the GH/IGF network in the liver, the primary tissue in which this network is expressed to integrate growth, energetics, and metabolism, and in the muscle, a tissue that also contributes to sexual dimorphism in body mass and is responsive to sex-specific regulators such as androgens. Animals were euthanized by decapitation and the liver and muscles along the femur were immediately excised. These tissues were immediately placed in RNAlater (RNAlater Stabilization Solution, Thermofisher Scientific, Wlatham, MA) and then allowed to incubate for 24 h at 4°C prior to freezing at −80°C until RNA extraction.

### RNA extraction and library preparation

We followed a Trizol reagent protocol (Trizol LS Ragent, Solution, Thermofisher Scientific, Wlatham, MA) for RNA extraction. High-throughput RNAseq library preparation took place at the Georgia Genomics and Bioinformatics Center, and included quality control, library quantification, barcoding, and pooling for sequencing on the Illumina Nextseq platform (Illumina, Inc., San Diego, CA, USA). For brown anoles, we sequenced a total of 96 samples (including samples not in the current study), yielding an average of 13.9 million reads per sample. For slender anoles, we sequenced a total of 96 samples (including samples not in the current study), which yielded a total of 1.6 million reads per sample.

### Data processing, read mapping, and selection of candidate genes

Reads were demultiplexed by barcodes, and the resulting data was trimmed and quality filtered using Trimmomatic version 0.36 (Bolger et al. 2014). We mapped cleaned reads to the green anole (*Anolis carolinensis*) transcriptome because it is a close relative (and congener) of both of our study species with a well-annotated and published genome (AnoCar2.0v2, Alfoldi et al. 2011). Reads were mapped to the *A.carolinensis* transcriptome using the program BWA version 0.7.13 (Bolger et al. 2014) and the MEM algorithm. We used the Samtools version 1.8 (Li et al. 2009) toolkit to convert file formats, sort alignments, index sorted files, merge files, and count transcripts for mapped genes. We excluded any samples with fewer than 800,000 mapped reads, which did not exclude any brown anole samples but resulted in exclusion of six slender anole samples across tissues and sexes. Final sample size was five female and five male brown anoles for all tissues and ages. For slender anoles, sample size was six females and six males for the liver but eight females and six males for muscle tissue. Data were normalized with the edgeR package (Robinson et al. 2010) in the R statistical environment using trimmed mean of M-values normalization method (Robinson and Oshlack 2010).

### Statistical analyses

We initially focused on the expression of 15 genes of interest that are the primary genes in the GH/IGF network. We selected genes of interest in the GH/IGF network from our larger transcriptomic expression dataset, resulting in a dataset of read counts (counts per million) for both sexes and two tissues of both species of anole. To ensure robustness of our results, we took several analytical approaches to test for differences in sex bias between brown anoles and slender anoles. Prior to analysis, we excluded any gene where the mean expression of both sexes was less than 2 cpm in any given tissue (Table 2). We did this to avoid zero-inflation and a potential lack of biological relevance for genes with such low expression. This procedure resulted in the removal of *IGF-1R*, *IGFBP6*, and *IGF-2BP1* across all tissues, leaving eleven robustly expressed GH/IGF-1 network genes for statistical analysis (Table 1). We first tested whether gene expression values met the assumptions of general linear models. We then tested for normality using the Shapiro–Wilks test and log-normality using the Kolmogorov–Smirnov test. Because tests for normality can be very sensitive to trivial deviations from normality (Ghasemi and Zahediasl 2012), we used an alpha of 0.01 to determine if expression of a gene was significantly different from normality. Genes with expression values that were significantly non-normal (*P* < 0.01) were then log-transformed (after adding 1 to all values if expression was zero for any sample). Log-transformed genes did not differ significantly (*P* < 0.01) from normality.

**Table 1:** Gene transcript IDs and descriptions for the 11 genes in the GH/IGF1 growth regulatory network that were the focus of our analyses.

ENSEMBL ID	Gene	ENSEMBL Description
ENSACAT00000010700	*GHR*	growth hormone receptor [Source:HGNC Symbol;Acc:4263]
ENSACAT00000016563	*IGF1*	insulin-like growth factor 1 (somatomedin C) [Source:HGNC Symbol;Acc:5464]
ENSACAT00000009701	*IGF2*	insulin-like growth factor 2 (somatomedin A) [Source:HGNC Symbol;Acc:5466]
ENSACAT00000008062	*IGFBP1*	insulin-like growth factor binding protein 1 [Source:HGNC Symbol;Acc:5469]
ENSACAT00000004558	*IGFBP2*	insulin-like growth factor binding protein 2, 36kDa [Source:HGNC Symbol;Acc:5471]
ENSACAT00000008083	*IGFBP3*	insulin-like growth factor binding protein 3 [Source:HGNC Symbol;Acc:5472]
ENSACAT00000016203	*IGFBP4*	insulin-like growth factor binding protein 4 [Source:HGNC Symbol;Acc:5473]
ENSACAT00000000083	*IGFBP5*	insulin-like growth factor binding protein 5 [Source:HGNC Symbol;Acc:5474]
ENSACAT00000002051	*IGFBP7*	insulin-like growth factor binding protein 7 [Source:HGNC Symbol;Acc:5476]
ENSACAT00000008070	*IGF2BP2*	insulin-like growth factor 2 mRNA binding protein 2 [Source:HGNC Symbol;Acc:28,867]
ENSACAT00000013612	*IGF2BP3*	insulin-like growth factor 2 mRNA binding protein 3 [Source:HGNC Symbol;Acc:28,868]

First, we tested whether sex bias in expression of three canonical, highly expressed growth genes (*GHR, IGF-1, IGF-2*) differed between species using general linear models with a species effect, sex effect, and species-by-sex interaction. Here, the species effect accounts for any differences between species, which could be due to intrinsic biological differences between species or technical differences in study design and transcriptomic analysis. Importantly, we used the species-by-sex interaction to test whether sex differences in expression differ between brown anoles and slender anoles while accounting for any main effects of species and sex. We conducted these analyses separately for each tissue (liver, muscle) and at two ontogenetic time points: (1) we compared juvenile, 4-month-old brown anoles to adult slender anoles as a time point where both species have minimal sexual size dimorphism, and (2) we compared adult, 12-month-old brown anoles to adult slender anoles as a time point when brown anoles are highly sexually dimorphic. We excluded the intermediate, 8-month time point for brown anoles from our *a priori* tests for sex-by-species interactions, but we report analyses of these 8-month data for descriptive purposes (below). 

Second, we compared multivariate expression of the entire GH/IGF network using separate principal components analysis (PCA) for each tissue. For the PCAs, we included 11 GH/IGF genes (Table 1) and expression data from both 4-month and 12-month-old brown anoles (excluding the 8-month time point) and from adult slender anoles. We then identified principal components describing variation in overall gene expression separately within each tissue. Principal components one (PC1) and two (PC2) together explained about two thirds of the total variance in gene expression in liver (68%) and muscle (66%), while other principal components each explained less than 15% of the total expression variance in either tissue. Therefore, we used PC1 and PC2 for downstream analyses. We compared PC1 and PC2 values using general linear models with a species effect, sex effect, and species-by-sex interaction. If the species-by-sex interaction was significant, we used Tukey's HSD to test for pairwise differences among all groups. 

Finally, we conducted exploratory analyses on a gene-by-gene basis for each of the 11 genes in the GH/IGF network (Table S1) using several approaches. First, we tested for difference in expression between the sexes within species using general linear models with sex as the independent variable and untransformed or log-transformed expression data as the dependent variable. Second, we tested for heteroscedasticity of expression data using the Levene test for equal variances, although we found similar results using other tests for equal variances (e.g., Bartlett test). If the expression of a particular gene had significantly unequal variance (*P* < 0.05), we also tested for a difference in expression between the sexes using the Welch's ANOVA, which allows for unequal standard deviations. To ensure that our results were robust to the assumptions of linear models, we also tested for differences in expression between the sexes using the non-parametric Wilcoxon test and evaluated significance using χ^2^ tests. We refer to these analyses as exploratory because we did not have specific *a priori* predictions for sex and species differences in the expression of most downstream genes in the GH/IGF pathway (e.g., IGFBPs), and because the large number of independent tests that we conducted on a gene-by-gene basis means that the unadjusted *P*-value (alpha = 0.05) for any given test should be interpreted accordingly. All statistical analyses were conducted in JMP v 16.0 (SAS corporation, Cary, NC, USA).

## Results

### Sexual dimorphism in body size

**Fig. 1:**
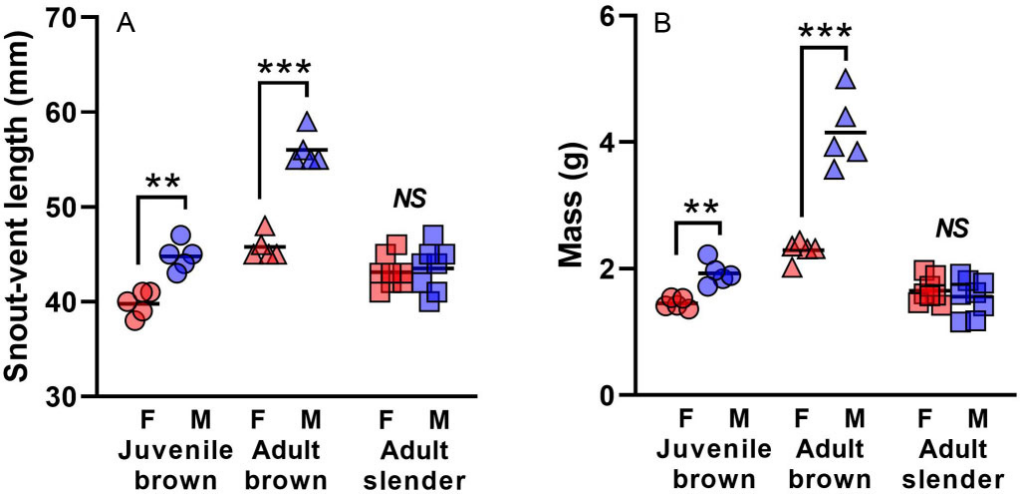
Size of juvenile (4 months) and adult (12 months) brown anoles (*n *= 5 for each sex and age group) and slender anoles (*n *= 8 of each sex) in length (A) and mass (C). Brackets and asterisks indicate significant differences between the sexes (*NS *= non-significant, *P < 0.05, **P < 0.001, ***P < 0.0001).

Male brown anoles were already significantly longer than females (*F*_1,9 _= 32.05, *P *= 0.0005), and more massive (*F*_1,9 _= 27.57, *P *= 0.0008) by 4 months of age (Fig. 1). The magnitude of sexual dimorphism in body size increased substantially in terms of both length (*F*_1,9 _= 110.68, *P *< 0.0001) and mass (*F*_1,9 _= 50.82, *P *< 0.0001) by 12 months of age for brown anoles. The body size of 12 month-old male brown anoles (mean SVL = 56.0 mm, mean mass = 4.15 g) from the laboratory colony was very similar to that of adult males (mean SVL = 56.5 mm, mean mass = 4.3 g) in the source population of Great Exuma (Cox and Calsbeek 2009). However, 12-month-old females (mean SVL = 45.8 mm, mean mass = 2.29 g) were larger than females (mean SVL = 42.9 mm, mean mass = 1.8 g) from the wild source population (Cox and Calsbeek 2009). In contrast, adult slender anole males (mean SVL = 43.5 mm, mean mass = 1.56 g) did not differ significantly (SVL: *F*_1,15 _= 0.14, *P *= 0.72, mass: *F*_1,15 _= 0.61, *P *= 0.45) in body size from adult female slender anoles (mean SVL = 43.1 mm, mean mass = 1.65 g). The adult body size in our sample of slender anoles is very similar to previously published results for reproductively mature adult slender anoles (Andrews 1979; Andrews and Stamps 1994).

### Species-specific pattern of sex bias in expression of GHR and IGFs

**Fig 2.**
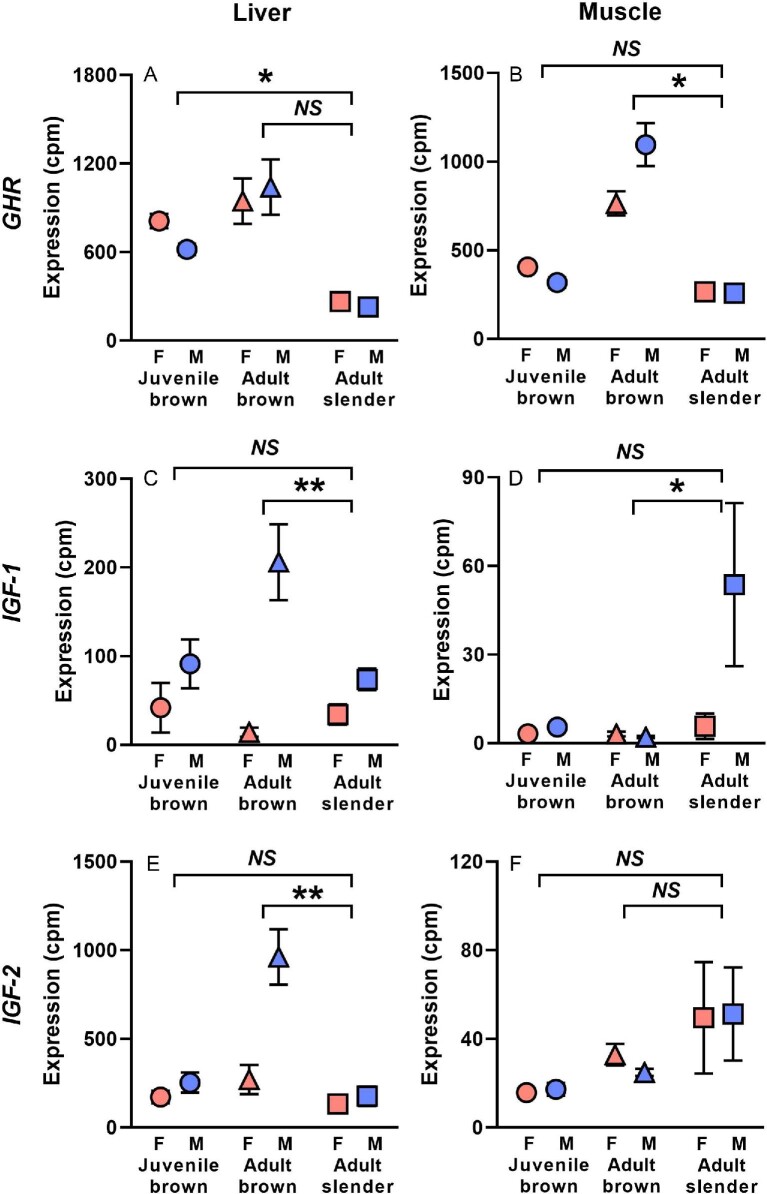
Expression in counts per million (cpm) of *GHR*, insulin like growth factor 1 (*IGF-1*), and insulin-like growth factor-2 (*IGF-2*) in the liver (A, C, E) and muscle (B, D, F) of brown anoles and slender anoles. For brown anoles, gene expression is shown separately for juveniles (4 months of age) and adults (12 months). Brackets and asterisks indicate significant sex-by-species interactions (*NS *= non-significant, *P < 0.05, **P < 0.001, ***P < 0.0001, see Table 2).

When comparing the expression of *GHR, IGF-1*, and *IGF-2* between juvenile (4-month-old) brown anoles and adult slender anoles, we only found one significant sex-by-species interaction across six comparisons (Table 2; Fig. 2). Specifically, expression of *GHR* was moderately female-biased in juvenile brown anoles, but only weakly female-biased in slender anoles (sex-by-species interaction: F_3,__18_ = 4.76; *P *= 0.043; Fig. 2A). *GHR* expression was not strongly sex-biased in muscle for either species (Fig. 2B). *IGF-1* expression was slightly male-biased in liver (sex: F_3,__18_ = 6.13; *P *= 0.024), but the magnitude of this sex bias was similar between species (sex-by-species interaction: F_3,__18_ = 2.37; *P *= 0.14; Fig. 2C). No sex effects or sex-by-species interactions were evident for *IGF-1* in muscle (Fig. 2D) or for *IGF-2* in either tissue (Fig. 2E and F).

**Table 2:** Results of general linear models with expression of each gene as the dependent variable, and species and sex as independent variables with interaction. Numerator degrees of freedom for the *F*-statistics are three for both tissues, while the denominator degrees of freedom are eighteen for liver and twenty for the muscle. An asterisk (*) by the gene name indicates that the expression data were log-transformed prior to analysis.

*4-month-old brown anoles vs. adult slender anoles*							
		Species		Sex		Sex by species	
Tissue	Gene	*F*	*P*	*F*	*P*	*F*	*P*
Liver	*GHR*	**174.64**	**<0.0001**	**10.41**	**0.0047**	**4.76**	**0.0426**
	IGF-1*	0.03	0.8737	**6.13**	**0.0235**	0.05	0.8238
	*IGF-2*	1.40	0.2518	1.63	0.2186	0.16	0.6942
	*IGFBP2*	**67.98**	**<0.0001**	0.44	0.5168	2.37	0.1408
	*IGFBP3*	**17.68**	**0.0005**	0.07	0.7894	0.13	0.7212
	*IGFBP4*	1.61	0.2208	0.32	0.5785	3.28	0.0870
Muscle	*GHR*	**15.06**	**0.0009**	3.38	0.0809	2.36	0.1403
	*IGF-1*	1.72	0.2049	1.69	0.2089	1.39	0.2516
	*IGF-2*	3.02	0.0976	0.01	0.9359	0.01	0.9948
	*IGFBP2*	0.01	0.9726	0.32	0.5782	1.60	0.2199
	*IGFBP3*	1.60	0.2205	2.98	0.0998	**8.79**	**0.0077**
	*IGFBP4*	0.71	0.4106	0.48	0.4983	0.35	0.5631
*12-month-old brown anoles vs. adult slender anoles*							
		Species		Sex		Sex by species	
Tissue	Gene	*F*	*P*	*F*	*P*	*F*	*P*

Liver	*GHR*	**211.74**	**<0.0001**	0.32	0.5815	1.34	0.2616
	*IGF-1*	**6.77**	**0.018**	**28.68**	**<0.0001**	**12.40**	**0.0024**
	*IGF-2*	**27.17**	**<0.0001**	**17.20**	**0.0006**	**13.43**	**0.0018**
	*IGFBP2*	**84.50**	**<0.0001**	**15.15**	**0.0011**	**23.83**	**0.0001**
	*IGFBP3*	**19.07**	**0.0004**	0.58	0.4566	1.77	0.2003
	IGFBP4*	**10.64**	**0.0430**	1.63	0.2185	0.77	0.3909
Muscle	*GHR*	**118.76**	**<0.0001**	**6.92**	**0.016**	**7.16**	**0.0121**
	IGF-1*	4.05	0.0579	3.43	0.0788	**5.47**	**0.0299**
	IGF-2*	0.47	0.5028	0.06	0.8135	0.39	0.5411
	*IGFBP2*	0.52	0.4792	0.10	0.7583	2.18	0.1552
	*IGFBP3*	**119.81**	**<0.0001**	**12.75**	**0.0019**	0.08	0.7767
	IGFBP4*	**4.88**	**0.0390**	1.95	0.1775	0.01	0.9752

By contrast, when comparing the expression of these same genes between adult (12-month-old) brown anoles and adult slender anoles, we found significant sex-by-species interactions in four of six comparisons (Table 2; Fig. 2). Expression of *GHR* in liver did not differ by sex in either species (Fig. 2A), but its expression in muscle was strongly male-biased in adult brown anoles and unbiased in adult slender anoles (F_3,__20_ = 2.37; *P *= 0.14; Fig. 2B). In the liver, expression of insulin-like growth factors was more strongly male-biased in adult brown anoles than in adult slender anoles for both *IGF-1* (sex-by-species interaction: F_3,__18_ = 12.40; *P = *0.004; Fig. 2C) and *IGF-2* (sex-by-species interaction: *F_3,__18_ *= 13.43; *P *=* *0.002; Fig. 2E). Levels of *IGF-1* and *IGF-2* expression were much lower in muscle (Fig. 2D and F), where expression of *IGF-1* was similarly low in male and female adult brown anoles, but male-biased in adult slender anoles (sex-by-species interaction: F_3,__20_ = 5.47; *P *= 0.030; Fig. 2D).

### Species-specific pattern of sex bias in expression of the entire GH/IGF network

**Fig. 3:**
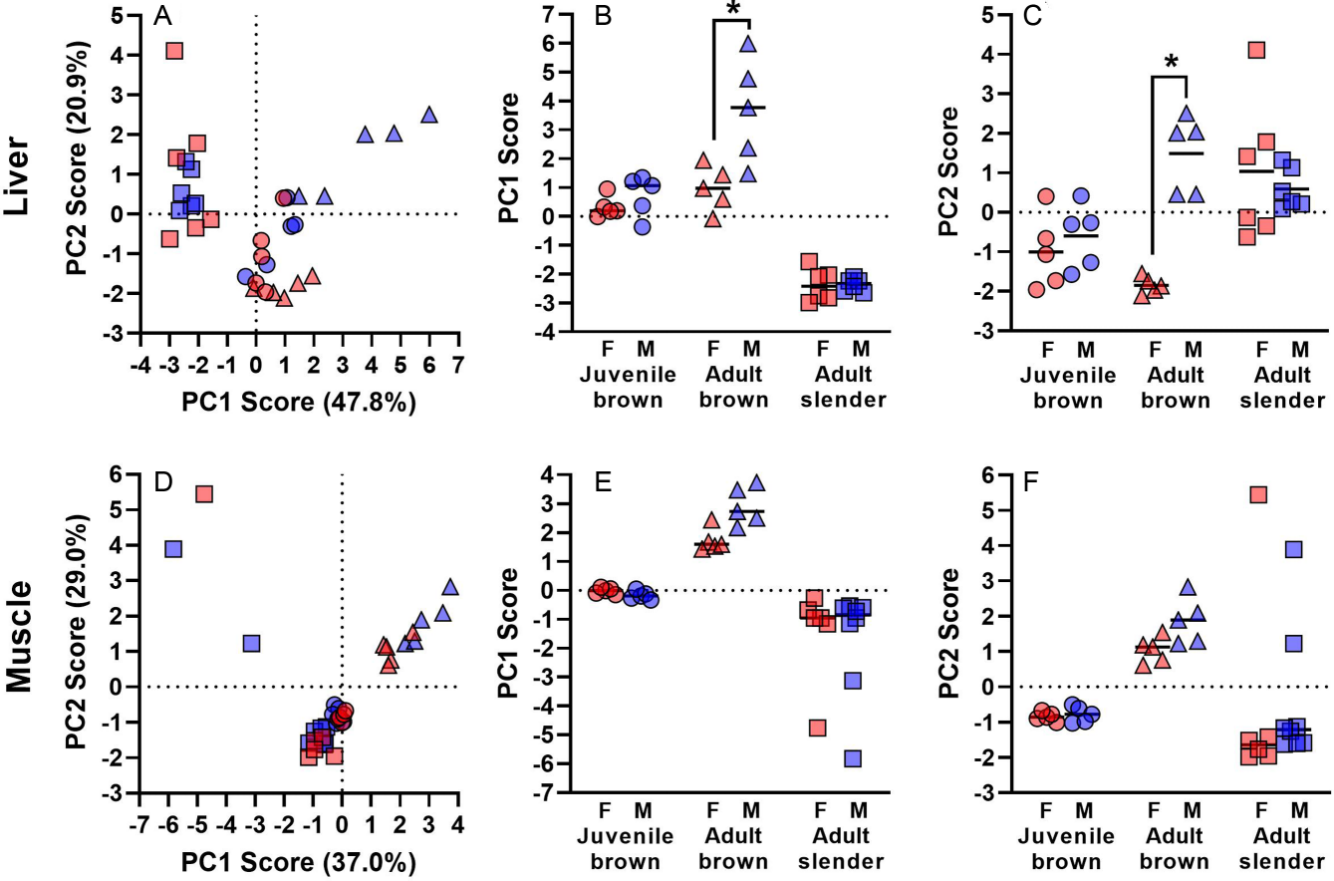
First and second principal component scores describing expression of 11 genes in the GH/IGF network in the liver (A) and muscle (D). Principal components 1 and 2 for each species and age point for the liver (B–C) and muscle (E–F). Brackets and asterisks indicate significant (*P* < 0.05) differences between the sexes within each species and age based upon Tukey's HSD.

When using PCA to reduce the dimensionality of expression data, we found that the major axes of GH/IGF network expression in both liver and muscle were consistently sex-biased in adult (12-month-old) brown anoles, but not in juvenile (4-month-old) brown anoles or slender anoles. For the liver, principal components 1 and 2 explained 47.8% and 20.9% of the variance in GH/IGF network-wide expression, respectively (Table S2), with PC1 primarily separating data by species (Fig. 3A). PC1 for liver was strongly positively correlated with the expression of most genes in the GH/IGF network, including *GHR, IGF-1, IGF-2,* and most binding proteins, but weakly negatively correlated with the expression of several other binding proteins (Table S2). PC2 for liver was positively correlated with the expression of *IGF-1, IGF-2,* and most binding proteins, but weakly negatively correlated with the expression of *GHR* and several other binding proteins. We found that both PC1 and PC2 values differed significantly between the sexes for adult brown anoles, but not for adult slender anoles or juvenile brown anoles (Fig. 3B and C). When comparing adult (12-month-old) brown anoles and adult slender anoles (Fig. 3B and C), liver PC1 values varied significantly based upon sex, species, and a sex-by-species interaction (Table 3). Adult male and female brown anoles differed significantly in liver PC1 values in a pairwise comparison (Tukey's HSD, *P* < 0.05), but the sexes did not differ significantly in PC1 values in slender anoles. In contrast, when comparing juvenile (4-month-old) brown anoles and adult slender anoles (Fig. 3B and C), liver PC1 values varied significantly only based upon species, but not sex or a sex-by-species interaction (Table 3). Similar to liver PC1, (Fig. 3B and C), liver PC2 values varied significantly based upon sex, species, and sex-by-species interaction when comparing adult (12-month-old) brown anoles and adult slender anoles (Table 3). Adult male and female brown anoles differed significantly in liver PC2 values in a pairwise comparison (Tukey's HSD, *P* < 0.05), but the sexes did not differ significantly in PC2 values in slender anoles. In contrast, (Fig. 3B and C), liver PC2 values varied significantly only based upon species, but not sex or a sex-by-species interaction when comparing juvenile (4-month-old) brown anoles and adult slender anoles (Table 3).

**Table 3:** Results of general linear models with PC1 and PC2 values as the dependent variable, and species and sex as independent variables with interaction. Separate principal component analyses of expression of the entire GH/IGF network were used to generate PC1 and PC2. Numerator degrees of freedom for the *F*-statistics are three for both tissues, while the denominator degrees of freedom are eighteen for liver and twenty for the muscle.

*4-month-brown anoles vs. adult slender anoles*							
		Species		Sex		Sex by species	
Tissue	Variable	*F*	*P*	*F*	*P*	*F*	*P*
Liver	*PC1*	**188.25**	**<0.0001**	0.89	0.3569	0.87	0.3648
	*PC2*	**10.80**	**0.0041**	0.01	0.9686	0.74	0.3998
Muscle	*PC1*	**6.64**	**0.0180**	0.12	0.7378	0.01	0.9490
	*PC2*	0.1908	0.6669	0.02	0.9048	0.01	0.9672
*12-month-brown anoles vs. adult slender anoles*							
		Species		Sex		Sex by species	
Tissue	Variable	*F*	*P*	*F*	*P*	*F*	*P*

Liver	*PC1*	**125.47**	**<0.0001**	**10.40**	**0.0047**	**10.35**	**0.0048**
	*PC2*	**4.50**	**0.0482**	**9.60**	**0.0062**	**16.38**	**0.0008**
Muscle	*PC1*	**43.51**	**<0.0001**	0.6520	0.4289	1.44	0.2440
	*PC2*	**5.92**	**0.0245**	0.36	0.5573	0.19	0.6672

For muscle, PC1 and PC2 explained 37.0% and 29.0% of the variance in GH/IGF network-wide expression, respectively (Table S2), with PC1 again separating data by species (Fig. 3D). PC1 for muscle was strongly positively correlated with the expression of *GHR* and several binding proteins*,* but negatively correlated with the expression of *IGF-1, IGF-2,* and several other binding proteins (Table S2). PC2 for muscle was strongly positively correlated with the expression of *GHR*, *IGF-1, IGF-2,* and most binding proteins, but weakly negatively correlated with the expression of several binding proteins, similar to loadings for PC1 in liver (Table S2). Muscle PC1 and PC2 values varied significantly between species, but not between sexes or with respect to the sex-by-species interaction either when comparing adult (12-month-old) or juvenile (4-month-old) brown anoles to adult slender anoles (Table 3, except for PC2 in the juvenile comparison). Although there was no sex-by-species interaction, there are sex differences in PC1 and PC2 values in the muscle for adult (12-month-old) brown anoles, but not adult slender anoles (Fig. 3E and F).

### Exploratory analyses of GH/IGF network genes

Generally, we found that both body size (Fig S1) and expression of *GHR*, *IGF-1*, and *IGF-2* in the liver and muscle at 8-months of age in brown anoles was intermediate in sex-biased expression to anoles of 4- and 12-months of age (Fig S2, Table S1). Several insulin-like growth factor binding proteins (*IGFBP*s) exhibited sex-biased expression in a given species, tissue, and/or age (Table S1), and patterns of sex-biased expression often differed between brown and slender anoles (Fig S3). In contrast, patterns of sex bias in insulin-like growth factor 2 binding proteins (*IGF2BP*s) differed between brown and slender anoles for the liver, but not for muscle tissue (Fig S4, Table S1).

## Discussion

Sex bias in expression of the entire GH/IGF network and key GH/IGF genes in the liver and muscle did not generally differ between slender anoles, which are sexually monomorphic in body size, and juvenile brown anoles, which are just beginning to develop sexual dimorphism in size. In contrast, sex bias in the expression of the entire GH/IGF network and key upstream GH/IGF genes in the liver and muscle differed sharply between sexually monomorphic adult slender anoles and adult brown anoles, which have substantial male-biased sexual size dimorphism. These results suggest that species-specific patterns of expression in the GH/IGF network are important for establishing differences in the direction and magnitude of sexual size dimorphism between species. More broadly, our results imply that evolutionary shifts in the sex-biased expression of regulatory networks such as the GH/IGF network could facilitate the evolution of sexual dimorphism.

Several caveats to our study bear mention. First, we sampled brown anoles from a captive breeding colony, whereas we sampled slender anoles from a wild population, which could conceivably lead to species differences in gene expression. However, brown anoles in this study were only three generations removed from the wild and reached similar body sizes to those in the wild source population (Cox and Calsbeek 2009), suggesting that growth divergence between the sexes that led to sexual dimorphism emerges in both environments. Second, we prepared and sequenced transcriptomic libraries at different times and at different sequencing depths, which could create differences in the magnitude and variability of gene expression between species. Although this means that the magnitude of expression of individual genes might not be directly comparable between species, we can still use sex-by-species interactions to gain insight into comparative patterns of sex-biased expression in the GH/IGF network while controlling for the overall statistical effect of species.

Previous work in lizards has found both male-biased hepatic *IGF-1* expression in male-larger brown anoles and female-biased hepatic *IGF-1* expression in female-larger eastern fence lizards (Cox et al. 2017b; Duncan et al. 2020). Interestingly, we found that expression of hepatic *IGF-1* was much less male-biased in sexually monomorphic adult slender anoles, relative to sexually dimorphic adult brown anoles. It is worth noting that IGF-1 has an important role in responding to nutrients and modulating metabolism (Duncan et al. 2020), which could also explain why the expression of *IGF-1* might differ between brown anoles and slender anoles. Similarly, *IGF-2* was male-biased in expression adult brown anoles, while it was not sex-biased in expression in the liver of slender anoles. Most functional research on *IGF-2* has been done in mammalian systems, where it is often only expressed prenatally (Wolf et al. 1998; Constância et al. 2002), and the function of IGF-2 in anole lizards is not known (Beatty and Schwartz 2020). However, our finding raises the intriguing possibility that sex-bias in expression of *IGF-2*, as well as *IGF-1*, might be important for the development and evolution of sexual dimorphism in body size in squamates.

These species-specific patterns of expression of the GH/IGF network suggest that there is an upstream modulator of expression that varies between species. In particular, the interaction between GH, which directly regulates expression of the *GH/IGF* network, and the steroid hormone testosterone, which is often male-biased in circulation and can be a bipotential regulator of growth in squamate reptiles, is likely to be important (Cox and John-Alder 2005; Cox et al. 2009a). Previous research has found that testosterone stimulates growth in species with male-biased sexual size dimorphism while inhibiting growth in species with female-biased sexual size dimorphism (Cox and John-Alder 2005; Cox et al. 2009a; Cox 2020). Indeed, testosterone stimulates *IGF-1* expression in male-larger brown anoles (Cox et al. 2017b), but can inhibit *IGF-1* expression in a species with female-biased sexual size dimorphism (Duncan 2011; Duncan et al. 2020). While slender anoles are monomorphic in body size, they are dimorphic in some ecological (perch height and width), morphological (dewlap size), and physiological (thermal tolerance) traits (Rosso et al. 2020; Logan et al. 2021; Neel et al. 2021), some of which can be impacted by testosterone in other species (Lovern et al. 2004; Cox et al. 2009b; Cox et al. 2015). It is conceivable that the relationship between testosterone and GH/IGF expression differs between brown anoles and slender anoles to produce the intraspecific variation in network expression and sexual dimorphism that we observed. Future research could leverage testosterone manipulations or studies of natural circulating variation in testosterone as an exploration of the mechanisms underlying alternate expression of the GH/IGF network in these two species.

The findings of our exploratory analyses of *IGF-* and *IGF-2* mRNA-binding protein expression should be interpreted with caution, given the sparse understanding of the role of these molecules in the GH/IGF network in anoles and lack of clear *a priori* hypotheses. However, we documented alternate patterns of expression for *IGFPBs* and *IGF2BPs* in both the muscle and the liver of brown and slender anoles. Neither brown nor slender anoles expressed *IGFBP6* or *IGF2BP1* at measurable levels, consistent with expression and predictions based upon molecular structure in other species for *IGFBP6* (McGaugh et al. 2015). We also found that *IGFBP1* was expressed at negligible levels across all age groups in both the liver and muscle of brown anoles but was expressed at relatively high levels in both tissues of slender anoles, which might suggest that this protein is not expressed in these tissues in brown anoles. Many IGF binding protein genes were sex-biased in expression in brown anoles (e.g., *IGFBP2, IGFBP4, IGFBP5, IGF2BP2,* and *IGF2BP3* in the liver), but were either unbiased or had the opposite pattern of sex bias in the slender anole. The specific functions of IGFBPs are not well understood outside of model systems (Clemmons 1993; Duan and Xu 2005; Duan et al. 2010; Allard and Duan 2018; Beatty and Schwartz 2020) and IGF2BPs have complex roles in cell biology that are not well understood (Beatty and Schwartz 2020). Our research hints that these components may play roles in the development and evolution of sexual dimorphism, but that additional research is needed to determine their functions in squamate reptiles.

Sexual size dimorphism varies substantially among anoles and other squamates (Stamps and Krishnan 1997; Butler et al. 2000), ranging from sexual monomorphism to highly sexually dimorphic and from male-larger to female-larger species (Shine 1978; Kratochvíl and Frynta 2002; Cox et al. 2003; Luo et al. 2012; Kahrl et al. 2016; Jiménez‐Arcos et al. 2017 ). Previous work has linked this variation in sexual size dimorphism to the variation in the impact of testosterone on growth (Cox and John-Alder 2005; Cox et al. 2009a). Our work highlights the potential role of the GH/IGF network in the evolution of sexual size dimorphism, as we found that adults of the dimorphic brown anole had greater sex bias in expression of the entire network and key GH/IGF genes. In contrast, the monomorphic slender anole had minimal or absent sex bias in the same network, similar to juveniles of the sexually dimorphic brown anole. Hence, evolution of sex-biased (or unbiased) expression of the GH/IGF network, could be linked to evolutionary variation in sexual size dimorphism in squamate reptiles.

## Supplementary Material

obac025_Supplemental_Files

## Data Accessibility

Raw read counts for both muscle and liver transcriptomes and normalized read-counts for GH/IGF genes are archived on figshare (https://doi.org/10.6084/m9.figshare.20324829.v1)
