# Supplementary material for: Species-Specific Expression of Growth-Regulatory Genes in 2 Anoles with Divergent Patterns of Sexual Size Dimorphism
Source: Integr Org Biol. 2022 Aug 9;4(1):obac025. doi: 10.1093/iob/obac025 (PMC9362763; doi:10.1093/iob/obac025)
Supplement: obac025_Supplemental_Files [file obac025_supplemental_files.zip › Supplementary_figure_legends.docx]

**Supplementary Figure Legends**

**Fig S1.** Size of brown anoles at 4, 8, and 12 months of age (*n*= 5 for each age group) in length (a) and mass (c), and size of adult slender anoles (*n*=8 of each age group) in length (b) and mass (d). Asterisks indicate when size differs significantly between the sexes. This figure differs from Fig. 1 because of the inclusion of the eight-month time point.

**Fig S2.** Expression in counts per million (cpm) of growth hormone receptor (GHR), insulin like growth factor 1 (IGF-1), and insulin-like growth factor-2 (IGF-2) in the liver and muscle of brown anoles and slender anoles. For brown anoles, gene expression is present for three different age points. Asterisks indicates genes are significantly sex-biased in expression (see Table 2). This figure differs from Fig. 2 because of the inclusion of the eight-month time point.

**Fig. S3.** Expression of insulin-like growth factor binding proteins (IGFBP1-5, IGFBP7) in the liver and muscle of brown anoles and slender anoles. For brown anoles, gene expression is present for three different age points. Asterisks indicates genes are significantly sex-biased in expression (see Table 2).

**Fig. S4.** Expression of insulin-like growth factor-2 binding proteins (IGF2BP2 and IGF2BP3) in the liver and muscle of brown anoles and slender anoles. For brown anoles, gene expression is present for three different age points. Asterisks indicates genes are significantly sex-biased in expression (see Table 2).
