# Supplementary material for: Species-Specific Expression of Growth-Regulatory Genes in 2 Anoles with Divergent Patterns of Sexual Size Dimorphism
Source: Integr Org Biol. 2022 Aug 9;4(1):obac025. doi: 10.1093/iob/obac025 (PMC9362763; doi:10.1093/iob/obac025)
Supplement: obac025_Supplemental_Files [file obac025_supplemental_files.zip › Table_S1.docx]

Table S1. Statistical results for tests of sex-biased expression of 11 genes in the growth regulatory network. An asterisk (*) by the gene name indicates that the data were log-transformed prior to analysis for that variable. Test statistics are F-statistics for linear models, χ2 for the Wilcoxon test, and F-statistics for Welch’s ANOVA. Welch’s ANOVA was only used for genes with expression data that failed the Levene test for equal variances. Dashes indicate when a statistical analysis was not used, either for genes that were not significantly heteroscedastic or for genes with average cpm less than 2.

|  |  |  | Linear model | | Wilcoxon test | | Welch's ANOVA | |
| --- | --- | --- | --- | --- | --- | --- | --- | --- |
|  | Gene | Direction of Sex Bias | Test Statistic | *P* | Test Statistic | *P* | Test Statistic | *P* |
| Brown anole | |  |  |  |  |  |  |  |
| *Liver, 12 mo* | *GHR* | Male | 0.6841 | 0.4322 | 0.5345 | 0.4647 | - | - |
|  | ***IGF1*** | **Male** | **19.6343** | **0.0022** | **6.8182** | **0.009** | **19.6343** | **0.0107** |
|  | *IGFBP1* | Male | 0.7072 | 0.4248 | 0.8836 | 0.3472 | - | - |
|  | ***IGFBP2*** | **Male** | **19.4978** | **0.0022** | **5.7709** | **0.0163** | **-** | **-** |
|  | *IGFBP3* | Female | 0.9005 | 0.3704 | 0.5345 | 0.4647 | - | - |
|  | ***IGFBP4*** | **Male** | **39.6608** | **0.0002** | **6.8182** | **0.009** | **-** | **-** |
|  | *IGFBP5* | Male | 3.278 | 0.1078 | 2.4545 | 0.1172 | - | - |
|  | *IGFBP7** | Male | 1.1308 | 0.3186 | 1.32 | 0.2506 | - | - |
|  | ***IGF2*** | **Male** | **15.2781** | **0.0045** | **5.7709** | **0.0163** | **-** | **-** |
|  | ***IGF2BP2*** | **Male** | **6.1782** | **0.0378** | **3.9382** | **0.0472** | **-** | **-** |
|  | ***IGF2BP3*** | **Female** | **14.1424** | **0.0055** | **6.8182** | **0.009** | **14.1424** | **0.0171** |
| *Muscle, 12 mo* | ***GHR*** | **Male** | **5.9649** | **0.0441** | **3.1527** | **0.0758** | - | - |
|  | *IGF1* | Female | 1.3483 | 0.279 | 0.5345 | 0.4647 | - | - |
|  | *IGFBP1* | Female | - | - | - | - | - | - |
|  | ***IGFBP2*** | **Male** | **3.8206** | **0.0864** | **3.9382** | **0.0472** | **-** | **-** |
|  | *IGFBP3* | Male | 4.5802 | 0.0648 | 3.1527 | 0.0758 | - | - |
|  | ***IGFBP4*** | **Female** | **12.9114** | **0.0071** | **6.8182** | **0.009** | **-** | **-** |
|  | *IGFBP5* | Male | 1.8839 | 0.2071 | 1.32 | 0.2506 | - | - |
|  | ***IGFBP7*** | **Female** | **13.9028** | **0.0058** | **4.8109** | **0.0283** | **-** | **-** |
|  | *IGF2* | Female | 2.4754 | 0.1543 | 1.32 | 0.2506 | - | - |
|  | *IGF2BP2* | Female | 0.3776 | 0.556 | 0.5345 | 0.4647 | - | - |
|  | *IGF2BP3* | Male | 3.9964 | 0.0806 | 2.4545 | 0.1172 | - | - |
| *Liver, 8 mo* | *GHR* | Male | 0.0001 | 0.9921 | 0.0982 | 0.754 | **-** | **-** |
|  | ***IGF1**** | Male | **56.3004** | **<0.0001** | **6.8182** | **0.009** | **27.7902** | **0.0055** |
|  | *IGFBP1* | Female | 1.0458 | 0.3364 | 0.2727 | 0.6015 | - | - |
|  | ***IGFBP2*** | Male | **37.7543** | **0.0003** | **6.8182** | **0.009** | **-** | **-** |
|  | *IGFBP3** | Female | 0.9006 | 0.3704 | 1.8436 | 0.1745 | - | - |
|  | ***IGFBP4*** | **Male** | **12.5479** | **0.0076** | **4.8109** | **0.0283** | **-** | **-** |
|  | ***IGFBP5*** | Male | **16.5586** | **0.0036** | **6.8182** | **0.009** |  |  |
|  | *IGFBP7* | **Male** | 0.0157 | 0.9034 | 0.0109 | 0.9168 | - | - |
|  | ***IGF2*** | Male | **13.3097** | **0.0065** | **6.8182** | **0.009** | **13.3097** | **0.0132** |
|  | ***IGF2BP2*** | **Male** | **17.2447** | **0.0032** | **6.8182** | **0.009** | **-** | **-** |
|  | *IGF2BP3* | Female | 1.2618 | 0.2939 | 1.8436 | 0.1745 | - | - |
| *Muscle, 8 mo* | *GHR* | Female | 0.6867 | 0.4313 | 0.8836 | 0.3472 | - | - |
|  | ***IGF1*** | **Male** | **6.488** | **0.0343** | **5.7709** | **0.0163** | **-** | **-** |
|  | *IGFBP1* | Female | 0.3317 | 0.5805 | 1.3528 | 0.2448 | - | - |
|  | *IGFBP2* | Male | 2.9826 | 0.1224 | 1.32 | 0.2506 | - | - |
|  | ***IGFBP3*** | **Female** | **6.272** | **0.0367** | **3.1527** | **0.0758** | **-** | **-** |
|  | *IGFBP4* | Female | 0.783 | 0.402 | 0.5345 | 0.4647 | 0.783 | 0.4132 |
|  | *IGFBP5* | Male | 0.6095 | 0.4575 | 0.8836 | 0.3472 | - | - |
|  | *IGFBP7** | Female | 0.6507 | 0.4432 | 0.5345 | 0.4647 | - | - |
|  | *IGF2* | Female | 0.0632 | 0.8079 | 0.0982 | 0.754 | - | - |
|  | *IGF2BP2* | Female | 0.455 | 0.519 | 1.32 | 0.2506 | - | - |
|  | *IGF2BP3* | Female | 3.3126 | 0.1062 | 2.4545 | 0.1172 | - | - |
| *Liver, 4 mo* | ***GHR*** | **Female** | **9.3472** | **0.0156** | **4.8109** | **0.0283** | **-** | **-** |
|  | *IGF1** | Male | 2.6201 | 0.1442 | 1.8436 | 0.1745 | - | - |
|  | *IGFBP1** | Female | 0.0501 | 0.8285 | 0.0109 | 0.9168 | - | - |
|  | *IGFBP2* | Male | 1.1689 | 0.3111 | 1.32 | 0.2506 | - | - |
|  | *IGFBP3* | Female | 0.0017 | 0.9679 | 0.0109 | 0.9168 | - | - |
|  | *IGFBP4* | Male | 1.5604 | 0.2469 | 0.8836 | 0.3472 | - | - |
|  | *IGFBP5* | Male | 0.5691 | 0.4623 | 0.8836 | 0.3472 | - | - |
|  | *IGFBP7* | Male | 0.0002 | 0.9879 | 0.2727 | 0.6015 | 0.002 | 0.988 |
|  | *IGF2* | Male | 1.4731 | 0.2595 | 1.32 | 0.2506 | - | - |
|  | *IGF2BP2* | Female | 0.9132 | 0.3672 | 0.5345 | 0.4647 | - | - |
|  | *IGF2BP3* | Female | 0.7935 | 0.399 | 1.32 | 0.2506 | - | - |
| *Muscle, 4 mo* | ***GHR*** | **Female** | **5.882** | **0.0415** | **3.9832** | **0.0472** | **-** | **-** |
|  | ***IGF1*** | **Male** | **5.9853** | **0.0402** | **3.9832** | **0.0472** | **-** | **-** |
|  | *IGFBP1* | Female | 2.937 | 0.1249 | 2.5155 | 0.1388 | - | - |
|  | *IGFBP2* | Male | 5.2221 | 0.0517 | 3.1527 | 0.0758 | 5.221 | 0.071 |
|  | *IGFBP3* | Female | 2.5125 | 0.1516 | 1.8436 | 0.1745 | - | - |
|  | *IGFBP4* | Female | 0.4219 | 0.5342 | 0.2727 | 0.6015 | - | - |
|  | *IGFBP5* | Male | 0.4857 | 0.5056 | 0.2727 | 0.6015 | - | - |
|  | ***IGFBP7**** | **Female** | **4.4827** | **0.0671** | **3.9382** | **0.0472** | **-** | **-** |
|  | *IGF2* | Male | 0.1755 | 0.6863 | 0.2727 | 0.6015 | - | - |
|  | *IGF2BP2* | Female | 0.5955 | 0.4625 | 0.5345 | 0.4647 | - | - |
|  | *IGF2BP3* | Male | 0.4567 | 0.5182 | 0.0109 | 0.9168 | - | - |
| Slender anole | |  |  |  |  |  |  |  |
| *Liver* | *GHR* | Female | 0.9203 | 0.36 | 0.641 | 0.4233 | - | - |
|  | ***IGF1*** | **Male** | **5.6345** | **0.039** | **4.33** | **0.0374** | **-** | - |
|  | *IGFBP1** | Female | 0.9939 | 0.3423 | 0.4103 | 0.5218 | 5.4657 | 0.4898 |
|  | *IGFBP2** | Female | 1.0077 | 0.3391 | 0.6643 | 0.4151 | - | - |
|  | ***IGFBP3*** | **Male** | **8.4433** | **0.0157** | **5.7692** | **0.0163** | **-** | **-** |
|  | *IGFBP4** | Female | 0.0491 | 0.8292 | 0.2316 | 0.6304 | 2.1626 | 0.1962 |
|  | *IGFBP5* | Female | - | - | - | - | - | - |
|  | *IGFBP7* | Female | 0.0496 | 0.8282 | 0.0256 | 0.8728 | 0.0496 | 0.8283 |
|  | *IGF2* | Male | 0.3823 | 0.5502 | 0.4103 | 0.5218 | - | - |
|  | *IGF2BP2* | Female | 0.1479 | 0.7086 | 0 | 1 | 0.1479 | 0.7143 |
|  | *IGF2BP3** | Female | 0.5814 | 0.4634 | 0.6892 | 0.4064 | - | - |
| *Muscle* | *GHR* | Female | 0.049 | 0.8286 | 0.0167 | 0.8973 | - | - |
|  | ***IGF1**** | **Male** | **6.5852** | **0.0247** | **3.7583** | **0.0525** | **2.944** | **0.1279** |
|  | *IGFBP1** | Male | 0.1757 | 0.6825 | 0.3041 | 0.5813 | - | - |
|  | *IGFBP2* | Female | - | - | - | - | - | - |
|  | ***IGFBP3*** | **Male** | **8.7478** | **0.012** | **6.0167** | **0.0142** | **-** | **-** |
|  | *IGFBP4** | Female | 0.7412 | 0.4062 | 1.3082 | 0.2527 | 0.4659 | 0.5194 |
|  | *IGFBP5* | Male | - | - | - | - | - | - |
|  | *IGFBP7* | Female | 1.4293 | 0.255 | 1.6814 | 0.1947 | - | - |
|  | *IGF2** | Male | 0.0591 | 0.8121 | 0.0167 | 0.8973 | - | - |
|  | *IGF2BP2* | Female | 3.0342 | 0.1071 | 2.8167 | 0.0933 | - | - |
|  | *IGF2BP3* | Male | - | - | - | - | - | - |
