# Supplementary material for: Species-Specific Expression of Growth-Regulatory Genes in 2 Anoles with Divergent Patterns of Sexual Size Dimorphism
Source: Integr Org Biol. 2022 Aug 9;4(1):obac025. doi: 10.1093/iob/obac025 (PMC9362763; doi:10.1093/iob/obac025)
Supplement: obac025_Supplemental_Files [file obac025_supplemental_files.zip › Table_S2.docx]

Table S2. Factor loadings for the first two principal components of gene expression in the GH/IGF network.

| **Tissue** | **Gene** | **Principal Component 1** | **Principal Component 2** |
| --- | --- | --- | --- |
| *Liver* | *GHR* | 0.888382 | -0.19242 |
|  | *IGF1* | 0.644837 | 0.588369 |
|  | *IGFBP1* | -0.4081709 | 0.592417 |
|  | *IGFBP2* | 0.8737461 | 0.071681 |
|  | *IGFBP3* | 0.5226549 | -0.4626 |
|  | *IGFBP4* | 0.7926819 | 0.4468 |
|  | *IGFBP5* | 0.8601548 | 0.161107 |
|  | *IGFBP7* | -0.5113664 | 0.369267 |
|  | *IGF2* | 0.8130979 | 0.384393 |
|  | *IGF2BP2* | -0.0828217 | 0.789551 |
|  | *IGF2BP3* | 0.7401258 | -0.45746 |
| *Muscle* | *GHR* | 0.7797263 | 0.533606 |
|  | *IGF1* | -0.615324 | 0.374268 |
|  | *IGFBP1* | -0.7087917 | 0.583493 |
|  | *IGFBP2* | -0.1181752 | 0.730051 |
|  | *IGFBP3* | 0.7310731 | 0.543853 |
|  | *IGFBP4* | -0.4675175 | 0.766168 |
|  | *IGFBP5* | 0.7575756 | 0.544973 |
|  | *IGFBP7* | -0.1782911 | -0.10731 |
|  | *IGF2* | -0.6697634 | 0.706532 |
|  | *IGF2BP2* | 0.275217 | -0.13748 |
|  | *IGF2BP3* | 0.8239333 | 0.429324 |
