# Supplementary figures and images for: Species-Specific Expression of Growth-Regulatory Genes in 2 Anoles with Divergent Patterns of Sexual Size Dimorphism
Source: Integr Org Biol. 2022 Aug 9;4(1):obac025. doi: 10.1093/iob/obac025 (PMC9362763; doi:10.1093/iob/obac025)

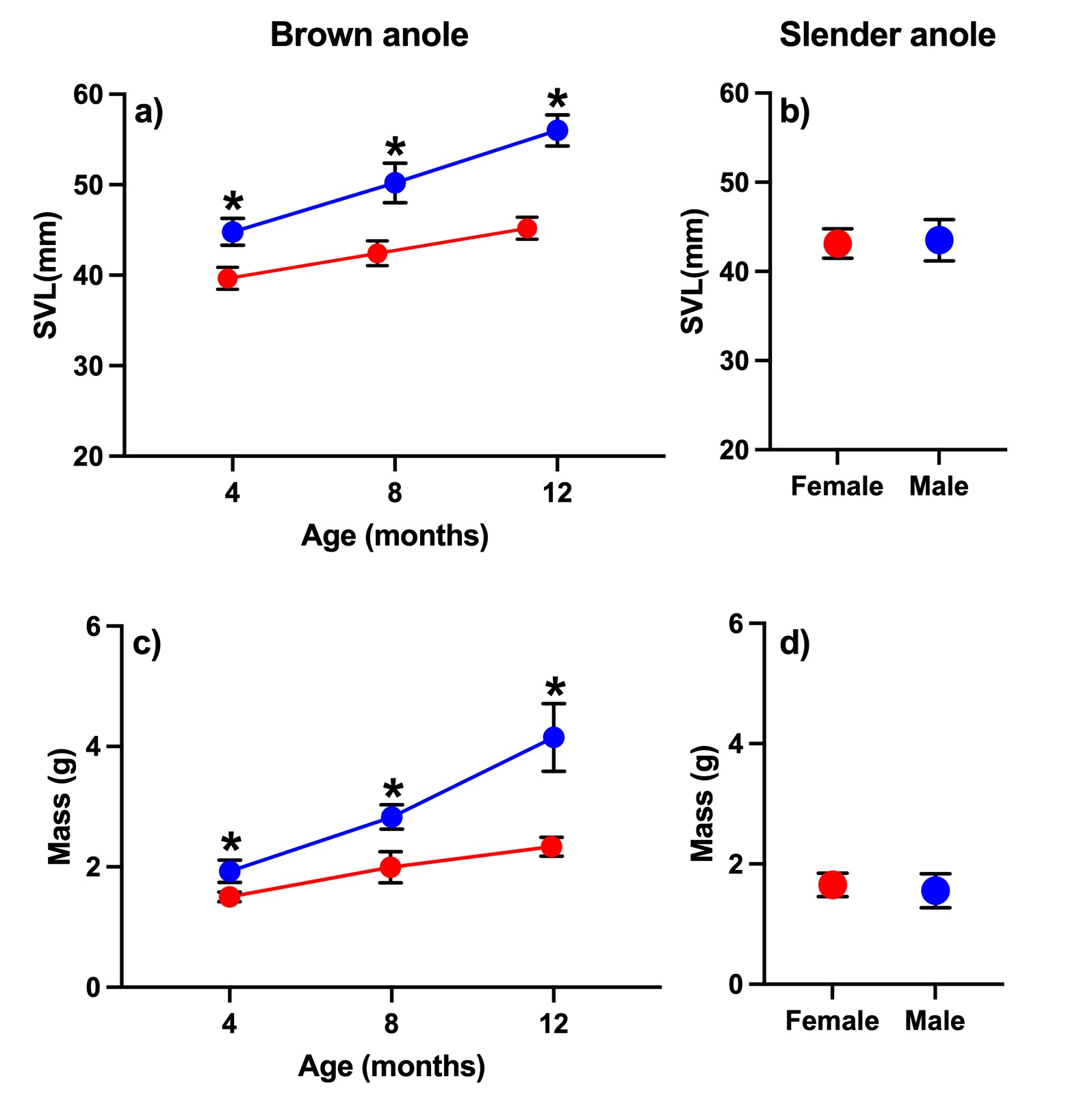

Supplement: obac025_Supplemental_Files [file obac025_supplemental_files.zip › FigS1size.jpg]

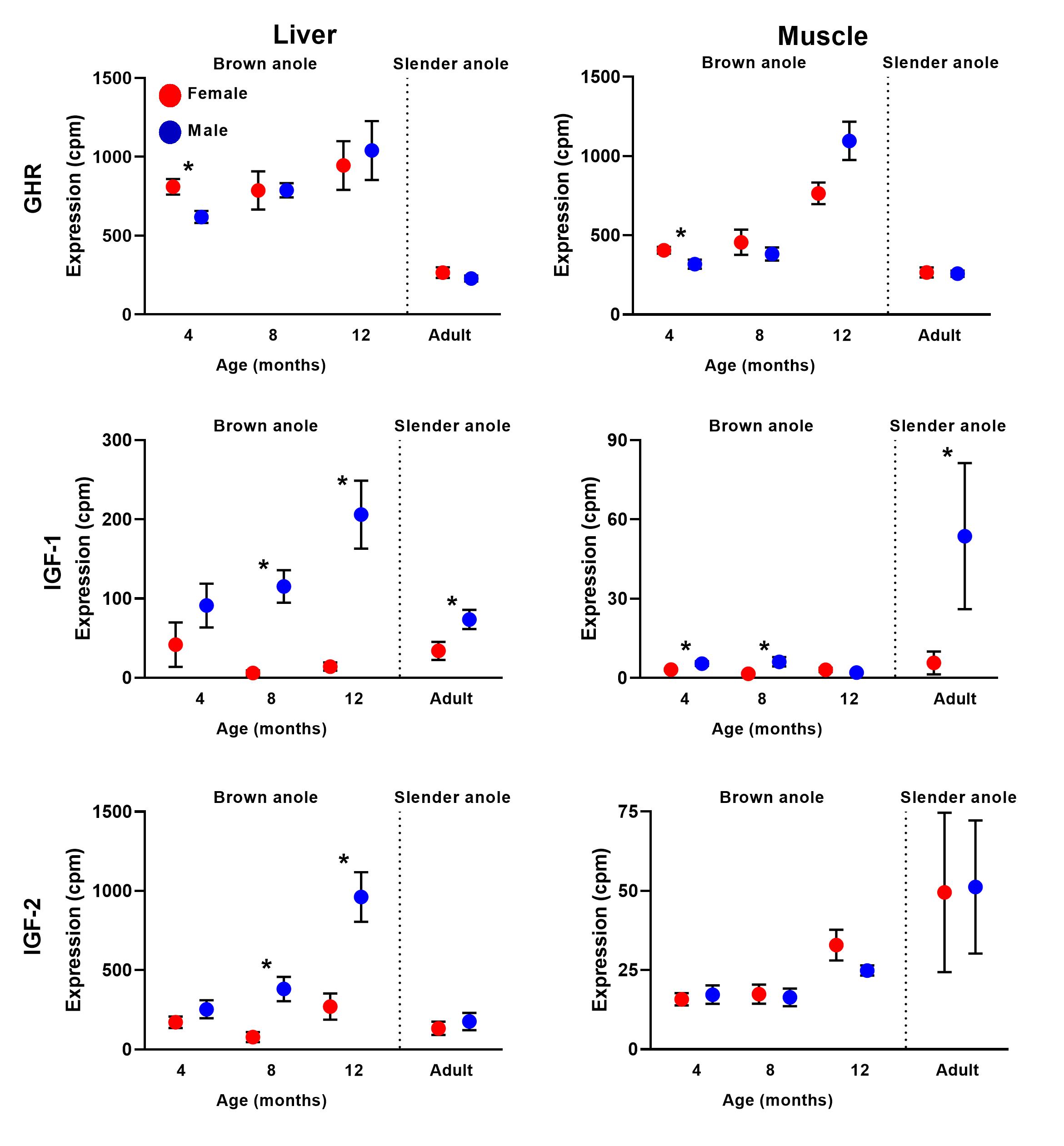

Supplement: obac025_Supplemental_Files [file obac025_supplemental_files.zip › FigS2_GHRIGF.jpg]

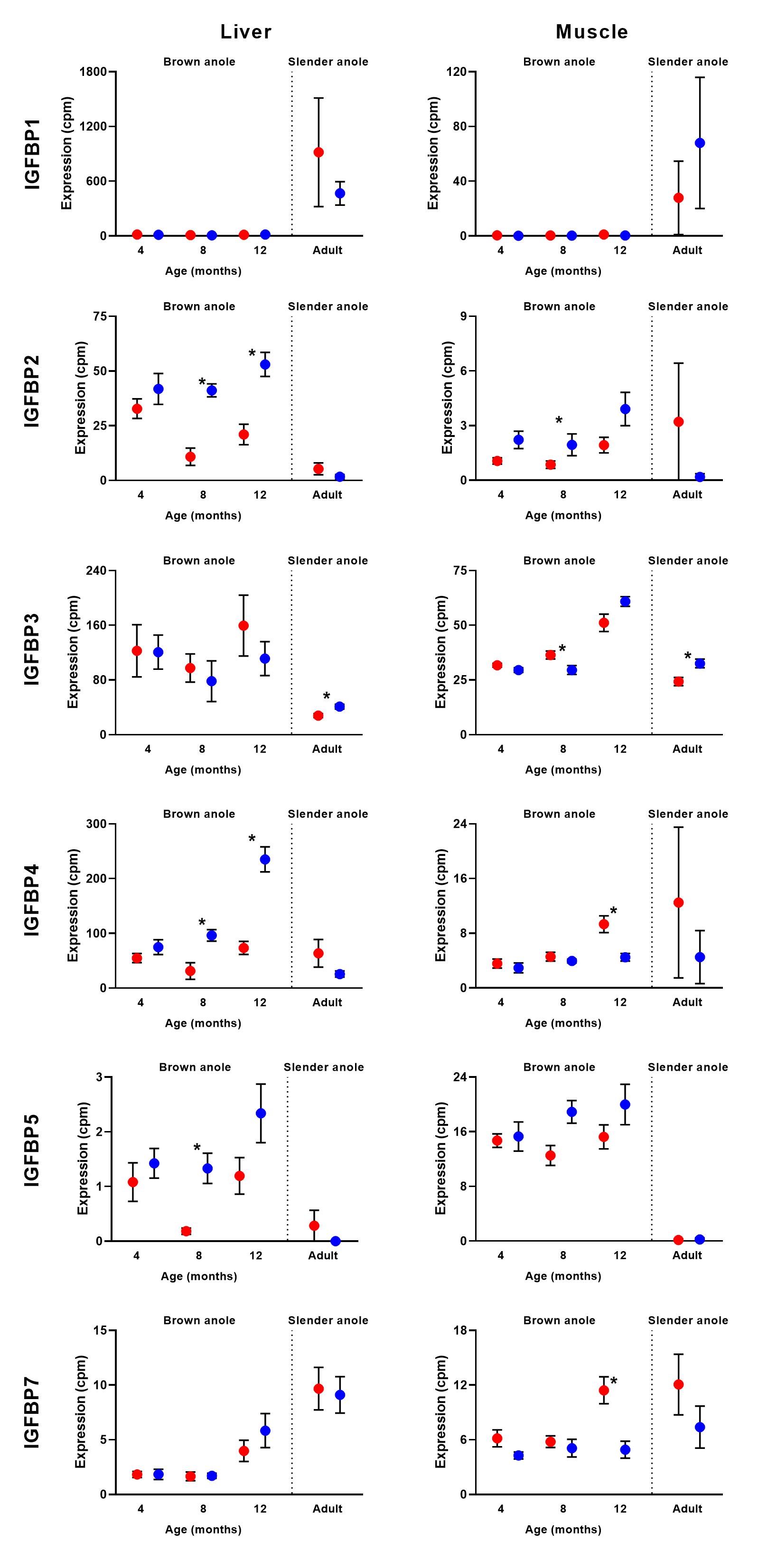

Supplement: obac025_Supplemental_Files [file obac025_supplemental_files.zip › FigS3_IGFBPs.jpg]

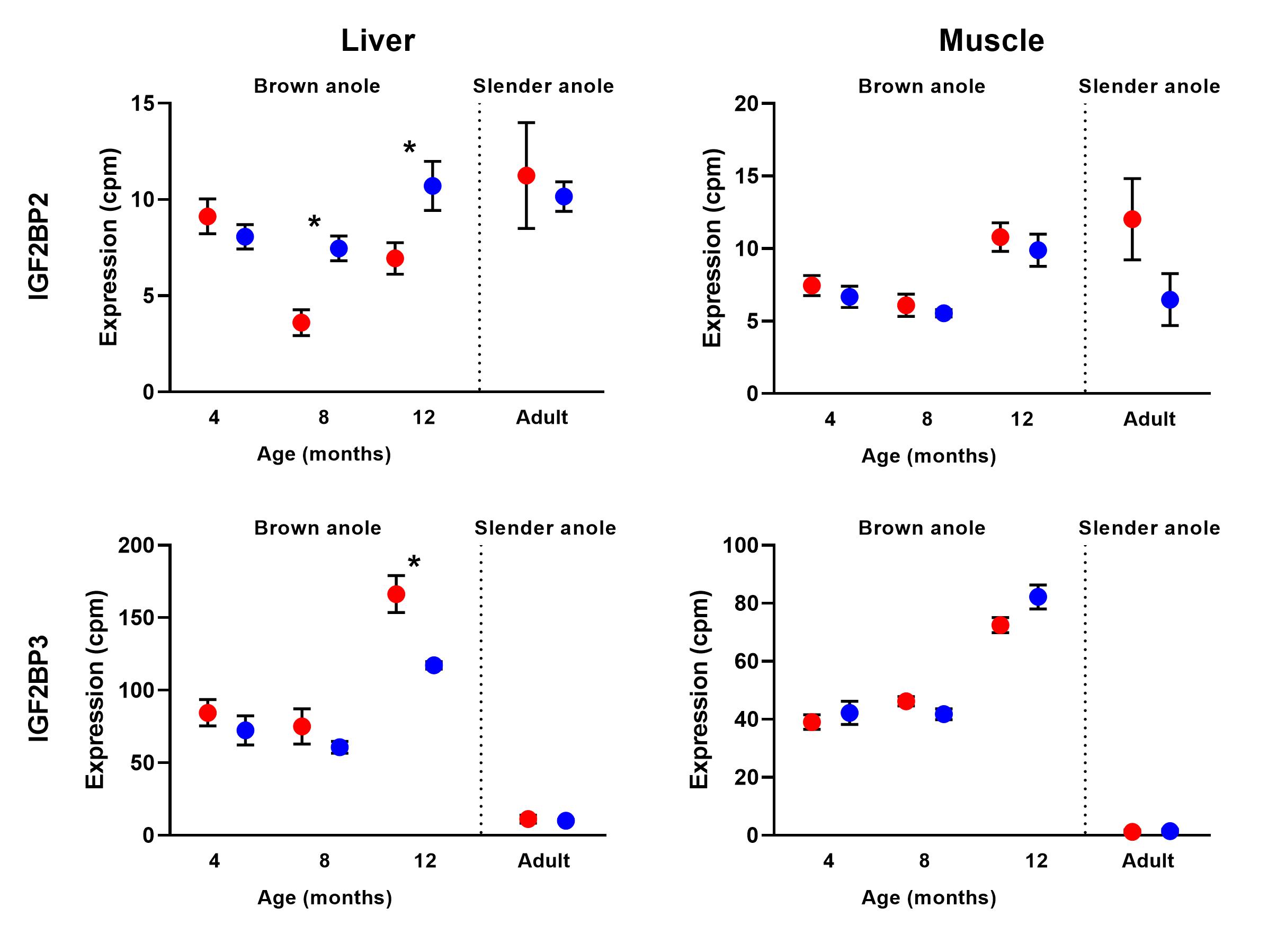

Supplement: obac025_Supplemental_Files [file obac025_supplemental_files.zip › FigS4_IGF2BPs.jpg]
